# 1,2-Dibenzoyl­hydrazine–dimethyl­formamide (3/1)

**DOI:** 10.1107/S1600536809016778

**Published:** 2009-05-14

**Authors:** Qing-Peng He, Hong-Gang Li, Guang-Bo Wang, Feng-Lian Fu, Ming-Shi Liu

**Affiliations:** a College of Chemistry and Chemical Engineering, Liaocheng University, Shandong 252059, People’s Republic of China; b Clinical Medicine Department, Weifang Medical University, Weifang, Shangdong, 261042, People’s Republic of China; c Shandong Wuxun High School, Guanxian, Shandong 252500, People’s Republic of China; d PetroChina Jinxi Branch Company (Bihai), 125001, People’s Republic of China

## Abstract

The title compound, 3C_14_H_12_N_2_O_2_·C_3_H_7_NO, was synthesized by reaction of benzoyl chloride with hydrazine hydrate under microwave irradition. The asymmetric unit comprises three 1,2-dibenzoyl­hydrazine mol­ecules and one dimethyl­formamide mol­ecule. The 1,2-dibenzoyl­hydrazine mol­ecules are linked by pairs of N—H⋯O hydrogen bonds into chains propagating along [010].

## Related literature

For background literature concerning microwave-assisted synthesis, see: Galema (1997 ▶). For the unsolvated crystal structure of 1,2-dibenzoyl­hydrazine, see: Shanmuga Sundara Raj *et al.* (2000 ▶).
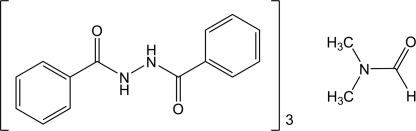


## Experimental

### 

#### Crystal data

3C_14_H_12_N_2_O_2_·C_3_H_7_NO
*M* *_r_* = 793.86
Triclinic, 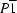

*a* = 10.7666 (12) Å
*b* = 11.4615 (13) Å
*c* = 18.100 (2) Å
α = 100.127 (2)°
β = 96.084 (2)°
γ = 108.382 (3)°
*V* = 2055.2 (4) Å^3^
*Z* = 2
Mo *K*α radiation
μ = 0.09 mm^−1^
*T* = 298 K
0.50 × 0.32 × 0.27 mm

#### Data collection

Bruker SMART APEX CCD diffractometer
Absorption correction: multi-scan (*SADABS*; Sheldrick, 1996 ▶) *T*_min_ = 0.957, *T*_max_ = 0.977
6846 measured reflections
4163 independent reflections
2487 reflections with *I* > 2σ(*I*)
*R*_int_ = 0.075
θ_max_ = 20.8°

#### Refinement

*R*[*F* ^2^ > 2σ(*F* ^2^)] = 0.120
*wR*(*F* ^2^) = 0.359
*S* = 1.14
4163 reflections
534 parameters
H-atom parameters constrained
Δρ_max_ = 0.66 e Å^−3^
Δρ_min_ = −0.52 e Å^−3^

### 

Data collection: *SMART* (Siemens, 1996 ▶); cell refinement: *SAINT* (Siemens, 1996 ▶); data reduction: *SAINT*; program(s) used to solve structure: *SHELXS97* (Sheldrick, 2008 ▶); program(s) used to refine structure: *SHELXL97* (Sheldrick, 2008 ▶); molecular graphics: *SHELXTL* (Sheldrick, 2008 ▶); software used to prepare material for publication: *SHELXTL*.

## Supplementary Material

Crystal structure: contains datablocks I, global. DOI: 10.1107/S1600536809016778/bi2365sup1.cif

Structure factors: contains datablocks I. DOI: 10.1107/S1600536809016778/bi2365Isup2.hkl

Additional supplementary materials:  crystallographic information; 3D view; checkCIF report

## Figures and Tables

**Table 1:** Hydrogen-bond geometry (Å, °)

*D*—H⋯*A*	*D*—H	H⋯*A*	*D*⋯*A*	*D*—H⋯*A*
N1—H1⋯O5^i^	0.86	2.04	2.846 (9)	156
N6—H6⋯O2^ii^	0.86	2.01	2.826 (9)	157
N2—H2⋯O3	0.86	2.00	2.800 (9)	155
N3—H3⋯O6	0.86	1.96	2.778 (10)	158
N4—H4⋯O1	0.86	1.92	2.743 (9)	160
N5—H5⋯O4	0.86	1.97	2.774 (9)	155

Symmetry codes: (i) 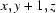; (ii) 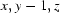.

**Fig. 1.**
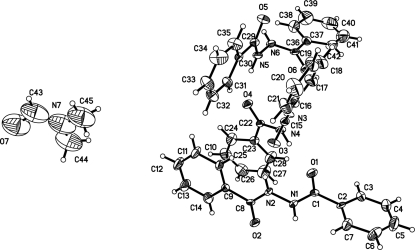
The content of asymmetric unit of the title compound showing the atomic numbering scheme and 30% probability displacement ellipsoids.The solvent molecular is omitted for clarity.

## supplementary crystallographic information

### Comment

In recent years, high-speed synthesis using microwave radiation has attracted considerable attention, and some important reviews in the study of microwave-assisted organic synthesis have been published (Galema, 1997). We describe in this paper a user-friendly microwave irradiation protocol for the synthesis of the title compound, and its crystal structure.

In the crystal structure, the asymmetric unit comprises three 1,2-dibenzoylhydrazine molecules and one dimethylformamide solvent molecule. The bond lengths and angles are normal and comparable to those in the unsolvated crystal structure of 1,2-dibenzoylhydrazine (Shanmuga Sundara Raj *et al.*, 2000). Molecules are linked by N—H···O hydrogen bonds between the amide H and carbonyl O atoms, forming ten-membered rings, into chains propagating along [010]. the dimethylformamide molecules lie between these chains.

### Experimental

Benzoyl chloride (0.5 mmol) and hydrazine hydrate (0.5 mmol) were mixed in a 50 ml flask. After microwave irradiation for 5 min at 275 W, then cooling to room temperature, the resulting mixture was washed with 10 ml water to yield a white product. The crude product was recrystallised from ethanol to afford the title compound as a crystalline solid. Elemental analysis calculated for C_45_H_43_N_7_O_7_: C 68.08, H 5.46, N 12.35%; found: C 68.24, H 5.68, N 12.28%.

### Refinement

All H atoms were placed in idealized positions (C—H = 0.93–0.96 Å, N—H 0.86 Å) and constrained to ride on their parent atoms, with *U*_iso_(H) = 1.2 or 1.5 *U*_eq_(C/N). The crystal diffracted relatively weakly and data are truncated to 1.00 Å resolution, with ca 60% of data observed at the 2σ(I) level. The structure is therefore of relatively low precision.

### Crystal data

**Table:**

3C_14_H_12_N_2_O_2_·C_3_H_7_NO	*Z* = 2
*M**_r_* = 793.86	*F*(000) = 836
Triclinic, *P*1	*D*_x_ = 1.283 Mg m^−^^3^
Hall symbol: -P 1	Mo *K*α radiation, λ = 0.71073 Å
*a* = 10.7666 (12) Å	Cell parameters from 1452 reflections
*b* = 11.4615 (13) Å	θ = 2.3–21.0°
*c* = 18.100 (2) Å	µ = 0.09 mm^−^^1^
α = 100.127 (2)°	*T* = 298 K
β = 96.084 (2)°	Needle, colourless
γ = 108.382 (3)°	0.50 × 0.32 × 0.27 mm
*V* = 2055.2 (4) Å^3^	

### Data collection

**Table:**

Bruker SMART APEX CCD diffractometer	4163 independent reflections
Radiation source: fine-focus sealed tube	2487 reflections with *I* > 2σ(*I*)
graphite	*R*_int_ = 0.075
φ and ω scans	θ_max_ = 20.8°, θ_min_ = 2.0°
Absorption correction: multi-scan (*SADABS*; Sheldrick, 1996)	*h* = −10→10
*T*_min_ = 0.957, *T*_max_ = 0.977	*k* = −11→11
6846 measured reflections	*l* = −10→18

### Refinement

**Table:**

Refinement on *F*^2^	Primary atom site location: structure-invariant direct methods
Least-squares matrix: full	Secondary atom site location: difference Fourier map
*R*[*F*^2^ > 2σ(*F*^2^)] = 0.120	Hydrogen site location: inferred from neighbouring sites
*wR*(*F*^2^) = 0.359	H-atom parameters constrained
*S* = 1.14	*w* = 1/[σ^2^(*F*_o_^2^) + (0.2*P*)^2^] where *P* = (*F*_o_^2^ + 2*F*_c_^2^)/3
4163 reflections	(Δ/σ)_max_ = 0.001
534 parameters	Δρ_max_ = 0.66 e Å^−^^3^
0 restraints	Δρ_min_ = −0.52 e Å^−^^3^

### Special details

**Table:**

Geometry. All e.s.d.'s (except the e.s.d. in the dihedral angle between two l.s. planes) are estimated using the full covariance matrix. The cell e.s.d.'s are taken into account individually in the estimation of e.s.d.'s in distances, angles and torsion angles; correlations between e.s.d.'s in cell parameters are only used when they are defined by crystal symmetry. An approximate (isotropic) treatment of cell e.s.d.'s is used for estimating e.s.d.'s involving l.s. planes.
Refinement. Refinement of *F*^2^ against ALL reflections. The weighted *R*-factor *wR* and goodness of fit *S* are based on *F*^2^, conventional *R*-factors *R* are based on *F*, with *F* set to zero for negative *F*^2^. The threshold expression of *F*^2^ > σ(*F*^2^) is used only for calculating *R*-factors(gt) *etc*. and is not relevant to the choice of reflections for refinement. *R*-factors based on *F*^2^ are statistically about twice as large as those based on *F*, and *R*- factors based on ALL data will be even larger.

### Fractional atomic coordinates and isotropic or equivalent isotropic displacement parameters (Å^2^)

**Table:**

	*x*	*y*	*z*	*U*_iso_*/*U*_eq_	
N1	0.1213 (7)	0.7581 (6)	0.2502 (4)	0.0367 (18)	
H1	0.0827	0.8132	0.2585	0.044*	
N2	0.1953 (7)	0.7353 (7)	0.3099 (4)	0.0373 (19)	
H2	0.1642	0.6671	0.3257	0.045*	
N3	0.1123 (6)	0.3584 (6)	0.2436 (4)	0.0377 (19)	
H3	0.0958	0.2827	0.2184	0.045*	
N4	0.2312 (6)	0.4518 (6)	0.2435 (4)	0.0364 (18)	
H4	0.2316	0.5089	0.2184	0.044*	
N5	0.1935 (7)	0.1264 (6)	0.3108 (4)	0.0368 (18)	
H5	0.2342	0.2062	0.3268	0.044*	
N6	0.2322 (7)	0.0597 (6)	0.2525 (4)	0.0354 (18)	
H6	0.2766	0.0117	0.2619	0.042*	
N7	0.380 (3)	0.619 (2)	0.8906 (19)	0.200 (10)	
O1	0.1675 (7)	0.6159 (6)	0.1659 (3)	0.0541 (18)	
O2	0.3604 (6)	0.9179 (6)	0.3238 (3)	0.0472 (17)	
O3	0.0421 (6)	0.4918 (6)	0.3186 (4)	0.0495 (17)	
O4	0.3487 (5)	0.3746 (6)	0.3198 (3)	0.0460 (16)	
O5	0.0327 (6)	−0.0487 (6)	0.3225 (3)	0.0491 (17)	
O6	0.1339 (7)	0.1356 (6)	0.1664 (3)	0.0550 (18)	
O7	0.5384 (19)	0.6803 (19)	1.0039 (13)	0.241 (8)	
C1	0.1106 (9)	0.6927 (8)	0.1791 (5)	0.036 (2)	
C2	0.0228 (9)	0.7136 (8)	0.1180 (5)	0.040 (2)	
C3	0.0276 (11)	0.6610 (10)	0.0446 (6)	0.062 (3)	
H3A	0.0873	0.6187	0.0359	0.074*	
C4	−0.0520 (15)	0.6694 (11)	−0.0146 (6)	0.077 (4)	
H4A	−0.0452	0.6346	−0.0638	0.093*	
C5	−0.1417 (14)	0.7274 (14)	−0.0044 (7)	0.082 (4)	
H5A	−0.1977	0.7307	−0.0462	0.099*	
C6	−0.1506 (11)	0.7816 (12)	0.0676 (8)	0.080 (4)	
H6A	−0.2132	0.8208	0.0753	0.095*	
C7	−0.0630 (10)	0.7770 (10)	0.1300 (6)	0.059 (3)	
H7	−0.0642	0.8173	0.1791	0.071*	
C8	0.3152 (9)	0.8189 (8)	0.3433 (4)	0.032 (2)	
C9	0.3875 (8)	0.7849 (8)	0.4068 (4)	0.034 (2)	
C10	0.3335 (9)	0.6761 (9)	0.4323 (5)	0.042 (2)	
H10	0.2486	0.6210	0.4099	0.050*	
C11	0.4055 (10)	0.6491 (11)	0.4910 (5)	0.058 (3)	
H11	0.3709	0.5739	0.5066	0.070*	
C12	0.5278 (11)	0.7334 (12)	0.5261 (6)	0.069 (3)	
H12	0.5746	0.7186	0.5676	0.083*	
C13	0.5791 (11)	0.8381 (12)	0.4996 (7)	0.079 (4)	
H13	0.6642	0.8930	0.5217	0.095*	
C14	0.5094 (9)	0.8667 (10)	0.4404 (5)	0.059 (3)	
H14	0.5459	0.9409	0.4242	0.071*	
C15	0.0250 (8)	0.3862 (9)	0.2823 (5)	0.034 (2)	
C16	−0.1008 (8)	0.2810 (8)	0.2787 (5)	0.037 (2)	
C17	−0.1390 (9)	0.1684 (10)	0.2308 (6)	0.061 (3)	
H17	−0.0848	0.1536	0.1962	0.073*	
C18	−0.2547 (12)	0.0737 (11)	0.2306 (8)	0.088 (4)	
H18	−0.2788	−0.0021	0.1949	0.106*	
C19	−0.3312 (12)	0.0886 (12)	0.2797 (7)	0.079 (4)	
H19	−0.4076	0.0225	0.2805	0.094*	
C20	−0.2992 (11)	0.2016 (14)	0.3301 (7)	0.096 (5)	
H20	−0.3558	0.2150	0.3635	0.115*	
C21	−0.1791 (10)	0.2973 (11)	0.3306 (6)	0.072 (3)	
H21	−0.1532	0.3726	0.3669	0.086*	
C22	0.3466 (8)	0.4540 (8)	0.2825 (5)	0.028 (2)	
C23	0.4680 (8)	0.5553 (8)	0.2778 (5)	0.035 (2)	
C24	0.5759 (9)	0.5837 (11)	0.3321 (6)	0.060 (3)	
H24	0.5733	0.5376	0.3697	0.073*	
C25	0.6887 (10)	0.6805 (12)	0.3313 (7)	0.075 (3)	
H25	0.7604	0.7021	0.3706	0.091*	
C26	0.7002 (11)	0.7455 (12)	0.2764 (8)	0.078 (4)	
H26	0.7796	0.8080	0.2755	0.093*	
C27	0.5919 (13)	0.7169 (12)	0.2219 (8)	0.090 (4)	
H27	0.5964	0.7615	0.1834	0.108*	
C28	0.4764 (10)	0.6236 (11)	0.2228 (6)	0.064 (3)	
H28	0.4027	0.6064	0.1856	0.077*	
C29	0.0926 (8)	0.0672 (9)	0.3427 (5)	0.034 (2)	
C30	0.0563 (8)	0.1458 (8)	0.4058 (4)	0.031 (2)	
C31	0.1238 (9)	0.2727 (9)	0.4328 (5)	0.043 (2)	
H31	0.1956	0.3129	0.4112	0.052*	
C32	0.0865 (10)	0.3404 (11)	0.4910 (6)	0.060 (3)	
H32	0.1310	0.4268	0.5074	0.072*	
C33	−0.0139 (11)	0.2833 (13)	0.5246 (6)	0.069 (3)	
H33	−0.0357	0.3295	0.5656	0.082*	
C34	−0.0833 (14)	0.1591 (14)	0.4992 (7)	0.090 (4)	
H34	−0.1546	0.1204	0.5217	0.108*	
C35	−0.0472 (10)	0.0886 (10)	0.4387 (6)	0.063 (3)	
H35	−0.0943	0.0029	0.4212	0.076*	
C36	0.2001 (9)	0.0699 (8)	0.1802 (5)	0.036 (2)	
C37	0.2514 (9)	0.0059 (8)	0.1209 (5)	0.040 (2)	
C38	0.3468 (10)	−0.0487 (10)	0.1344 (6)	0.060 (3)	
H38	0.3815	−0.0460	0.1842	0.072*	
C39	0.3899 (14)	−0.1055 (13)	0.0763 (9)	0.097 (4)	
H39	0.4526	−0.1435	0.0861	0.116*	
C40	0.342 (2)	−0.1079 (14)	0.0027 (10)	0.109 (5)	
H40	0.3763	−0.1423	−0.0373	0.131*	
C41	0.2433 (19)	−0.0595 (15)	−0.0117 (7)	0.103 (5)	
H41	0.2061	−0.0657	−0.0617	0.123*	
C42	0.2011 (12)	−0.0039 (10)	0.0458 (6)	0.065 (3)	
H42	0.1351	0.0300	0.0352	0.079*	
C43	0.410 (3)	0.596 (3)	0.962 (2)	0.211 (14)	
H43	0.3533	0.5326	0.9804	0.253*	
C44	0.486 (3)	0.704 (2)	0.8626 (15)	0.210 (11)	
H44A	0.4908	0.7890	0.8813	0.315*	
H44B	0.4685	0.6839	0.8079	0.315*	
H44C	0.5690	0.6944	0.8802	0.315*	
C45	0.252 (3)	0.556 (2)	0.8505 (16)	0.214 (12)	
H45A	0.2029	0.4992	0.8783	0.321*	
H45B	0.2558	0.5102	0.8015	0.321*	
H45C	0.2096	0.6168	0.8438	0.321*	

### Atomic displacement parameters (Å^2^)

**Table:**

	*U*^11^	*U*^22^	*U*^33^	*U*^12^	*U*^13^	*U*^23^
N1	0.046 (4)	0.032 (4)	0.037 (5)	0.022 (4)	−0.004 (3)	0.012 (4)
N2	0.047 (5)	0.030 (4)	0.035 (4)	0.014 (4)	−0.007 (4)	0.015 (3)
N3	0.032 (4)	0.018 (4)	0.053 (5)	−0.001 (4)	0.006 (4)	0.001 (3)
N4	0.028 (4)	0.024 (4)	0.055 (5)	0.003 (4)	0.005 (4)	0.017 (3)
N5	0.055 (5)	0.021 (4)	0.036 (4)	0.013 (4)	0.014 (4)	0.009 (3)
N6	0.055 (5)	0.029 (4)	0.032 (4)	0.021 (4)	0.017 (4)	0.012 (3)
N7	0.137 (19)	0.131 (18)	0.30 (3)	0.011 (15)	0.05 (2)	0.01 (2)
O1	0.076 (5)	0.049 (4)	0.050 (4)	0.038 (4)	0.008 (3)	0.016 (3)
O2	0.046 (4)	0.033 (4)	0.058 (4)	0.007 (3)	−0.005 (3)	0.021 (3)
O3	0.048 (4)	0.026 (4)	0.068 (4)	0.006 (3)	0.014 (3)	0.004 (3)
O4	0.038 (4)	0.038 (4)	0.060 (4)	0.006 (3)	0.001 (3)	0.025 (3)
O5	0.051 (4)	0.028 (4)	0.062 (4)	0.006 (3)	0.020 (3)	0.003 (3)
O6	0.086 (5)	0.049 (4)	0.043 (4)	0.043 (4)	0.005 (3)	0.011 (3)
O7	0.176 (15)	0.178 (17)	0.33 (2)	0.041 (14)	0.001 (15)	0.025 (16)
C1	0.048 (6)	0.021 (5)	0.038 (6)	0.006 (5)	0.002 (4)	0.011 (4)
C2	0.048 (6)	0.022 (5)	0.049 (6)	0.012 (5)	−0.008 (5)	0.016 (5)
C3	0.088 (8)	0.046 (7)	0.044 (7)	0.028 (6)	−0.015 (6)	−0.005 (5)
C4	0.113 (10)	0.069 (9)	0.042 (7)	0.032 (8)	−0.014 (7)	0.002 (6)
C5	0.089 (9)	0.095 (10)	0.057 (9)	0.025 (8)	−0.014 (7)	0.031 (7)
C6	0.065 (8)	0.096 (10)	0.103 (11)	0.051 (7)	0.012 (7)	0.044 (8)
C7	0.059 (7)	0.069 (8)	0.053 (6)	0.028 (6)	−0.002 (5)	0.018 (5)
C8	0.047 (6)	0.019 (5)	0.033 (5)	0.016 (5)	0.005 (5)	0.006 (4)
C9	0.037 (6)	0.034 (6)	0.033 (5)	0.017 (5)	−0.001 (4)	0.004 (4)
C10	0.040 (5)	0.046 (6)	0.035 (5)	0.012 (5)	−0.004 (4)	0.013 (5)
C11	0.054 (7)	0.068 (7)	0.055 (6)	0.016 (6)	−0.004 (5)	0.037 (6)
C12	0.066 (8)	0.083 (9)	0.062 (7)	0.026 (7)	−0.009 (6)	0.036 (7)
C13	0.068 (8)	0.068 (9)	0.087 (9)	0.014 (7)	−0.037 (7)	0.024 (7)
C14	0.046 (6)	0.052 (7)	0.064 (7)	0.004 (6)	−0.013 (6)	0.009 (6)
C15	0.029 (5)	0.026 (6)	0.045 (5)	0.006 (5)	−0.003 (4)	0.010 (5)
C16	0.026 (5)	0.037 (6)	0.039 (5)	0.002 (5)	−0.001 (4)	0.008 (5)
C17	0.031 (6)	0.045 (7)	0.084 (8)	−0.012 (5)	0.002 (5)	0.012 (6)
C18	0.069 (8)	0.056 (8)	0.100 (10)	−0.033 (7)	0.014 (8)	0.018 (7)
C19	0.056 (8)	0.068 (9)	0.079 (9)	−0.021 (7)	−0.003 (7)	0.019 (7)
C20	0.061 (8)	0.103 (12)	0.093 (9)	−0.022 (8)	0.033 (7)	0.021 (9)
C21	0.046 (7)	0.077 (9)	0.066 (7)	−0.004 (6)	0.008 (6)	−0.001 (6)
C22	0.026 (5)	0.023 (5)	0.036 (5)	0.008 (4)	0.004 (4)	0.010 (4)
C23	0.026 (5)	0.033 (5)	0.045 (5)	0.008 (4)	0.010 (5)	0.011 (5)
C24	0.034 (6)	0.075 (8)	0.065 (7)	0.005 (6)	0.006 (5)	0.027 (6)
C25	0.049 (7)	0.068 (8)	0.081 (8)	−0.013 (6)	0.003 (6)	0.010 (7)
C26	0.050 (8)	0.067 (9)	0.097 (9)	−0.005 (6)	0.018 (7)	0.013 (8)
C27	0.070 (9)	0.083 (10)	0.104 (10)	−0.006 (8)	0.012 (8)	0.053 (8)
C28	0.041 (6)	0.074 (8)	0.079 (7)	0.005 (6)	0.009 (5)	0.046 (7)
C29	0.038 (5)	0.029 (6)	0.039 (5)	0.015 (5)	0.007 (4)	0.010 (5)
C30	0.039 (5)	0.030 (6)	0.026 (5)	0.013 (4)	0.006 (4)	0.009 (4)
C31	0.045 (6)	0.042 (7)	0.040 (5)	0.015 (5)	0.010 (4)	0.001 (5)
C32	0.061 (7)	0.057 (7)	0.058 (7)	0.023 (6)	0.014 (6)	−0.004 (6)
C33	0.076 (8)	0.072 (9)	0.057 (7)	0.031 (7)	0.025 (6)	−0.009 (6)
C34	0.109 (10)	0.088 (11)	0.098 (9)	0.045 (9)	0.073 (8)	0.031 (8)
C35	0.067 (7)	0.054 (7)	0.070 (7)	0.015 (6)	0.040 (6)	0.012 (6)
C36	0.053 (6)	0.021 (5)	0.038 (6)	0.011 (5)	0.014 (5)	0.013 (4)
C37	0.045 (6)	0.035 (6)	0.038 (6)	0.007 (5)	0.013 (4)	0.008 (4)
C38	0.063 (7)	0.076 (8)	0.052 (6)	0.038 (6)	0.022 (5)	0.010 (6)
C39	0.116 (11)	0.104 (11)	0.093 (11)	0.063 (9)	0.048 (9)	0.017 (9)
C40	0.160 (15)	0.077 (11)	0.089 (13)	0.028 (11)	0.070 (11)	0.008 (9)
C41	0.165 (15)	0.091 (11)	0.060 (9)	0.050 (11)	0.024 (9)	0.022 (8)
C42	0.115 (9)	0.050 (7)	0.045 (7)	0.039 (7)	0.028 (7)	0.016 (5)
C43	0.16 (3)	0.13 (2)	0.31 (4)	0.020 (19)	0.04 (3)	0.00 (3)
C44	0.17 (2)	0.14 (2)	0.29 (3)	0.017 (17)	0.06 (2)	0.00 (2)
C45	0.15 (2)	0.14 (2)	0.30 (3)	0.001 (17)	0.02 (2)	0.01 (2)

### Geometric parameters (Å, °)

**Table:**

N1—C1	1.344 (10)	C17—C18	1.369 (14)
N1—N2	1.385 (9)	C17—H17	0.930
N1—H1	0.860	C18—C19	1.298 (16)
N2—C8	1.342 (10)	C18—H18	0.930
N2—H2	0.860	C19—C20	1.366 (17)
N3—C15	1.313 (10)	C19—H19	0.930
N3—N4	1.385 (9)	C20—C21	1.405 (15)
N3—H3	0.860	C20—H20	0.930
N4—C22	1.353 (10)	C21—H21	0.930
N4—H4	0.860	C22—C23	1.472 (12)
N5—C29	1.333 (10)	C23—C24	1.352 (12)
N5—N6	1.372 (9)	C23—C28	1.363 (12)
N5—H5	0.860	C24—C25	1.366 (14)
N6—C36	1.352 (10)	C24—H24	0.930
N6—H6	0.860	C25—C26	1.337 (16)
N7—C43	1.38 (3)	C25—H25	0.930
N7—C45	1.39 (3)	C26—C27	1.357 (15)
N7—C44	1.45 (3)	C26—H26	0.930
O1—C1	1.226 (10)	C27—C28	1.365 (15)
O2—C8	1.215 (9)	C27—H27	0.930
O3—C15	1.219 (10)	C28—H28	0.930
O4—C22	1.229 (9)	C29—C30	1.492 (11)
O5—C29	1.247 (10)	C30—C35	1.357 (12)
O6—C36	1.227 (10)	C30—C31	1.375 (11)
O7—C43	1.45 (3)	C31—C32	1.369 (13)
C1—C2	1.480 (12)	C31—H31	0.930
C2—C7	1.356 (13)	C32—C33	1.341 (14)
C2—C3	1.372 (13)	C32—H32	0.930
C3—C4	1.337 (14)	C33—C34	1.350 (16)
C3—H3A	0.930	C33—H33	0.930
C4—C5	1.345 (16)	C34—C35	1.408 (15)
C4—H4A	0.930	C34—H34	0.930
C5—C6	1.368 (16)	C35—H35	0.930
C5—H5A	0.930	C36—C37	1.450 (12)
C6—C7	1.412 (15)	C37—C42	1.381 (13)
C6—H6A	0.930	C37—C38	1.383 (13)
C7—H7	0.930	C38—C39	1.340 (15)
C8—C9	1.497 (12)	C38—H38	0.930
C9—C14	1.351 (12)	C39—C40	1.369 (19)
C9—C10	1.380 (12)	C39—H39	0.930
C10—C11	1.386 (12)	C40—C41	1.37 (2)
C10—H10	0.930	C40—H40	0.930
C11—C12	1.369 (14)	C41—C42	1.321 (18)
C11—H11	0.930	C41—H41	0.930
C12—C13	1.347 (15)	C42—H42	0.930
C12—H12	0.930	C43—H43	0.930
C13—C14	1.389 (14)	C44—H44A	0.960
C13—H13	0.930	C44—H44B	0.960
C14—H14	0.930	C44—H44C	0.960
C15—C16	1.488 (12)	C45—H45A	0.960
C16—C17	1.335 (13)	C45—H45B	0.960
C16—C21	1.354 (12)	C45—H45C	0.960
			
C1—N1—N2	118.9 (7)	C16—C21—C20	120.7 (11)
C1—N1—H1	120.5	C16—C21—H21	119.7
N2—N1—H1	120.5	C20—C21—H21	119.7
C8—N2—N1	120.5 (7)	O4—C22—N4	121.0 (7)
C8—N2—H2	119.7	O4—C22—C23	122.2 (7)
N1—N2—H2	119.7	N4—C22—C23	116.8 (8)
C15—N3—N4	119.7 (6)	C24—C23—C28	118.5 (9)
C15—N3—H3	120.1	C24—C23—C22	117.9 (8)
N4—N3—H3	120.1	C28—C23—C22	123.6 (8)
C22—N4—N3	120.8 (7)	C23—C24—C25	119.5 (10)
C22—N4—H4	119.6	C23—C24—H24	120.2
N3—N4—H4	119.6	C25—C24—H24	120.2
C29—N5—N6	119.9 (7)	C26—C25—C24	122.7 (11)
C29—N5—H5	120.1	C26—C25—H25	118.6
N6—N5—H5	120.1	C24—C25—H25	118.6
C36—N6—N5	119.5 (7)	C25—C26—C27	117.6 (11)
C36—N6—H6	120.2	C25—C26—H26	121.2
N5—N6—H6	120.2	C27—C26—H26	121.2
C43—N7—C45	118 (3)	C26—C27—C28	120.9 (12)
C43—N7—C44	118 (3)	C26—C27—H27	119.6
C45—N7—C44	125 (3)	C28—C27—H27	119.6
O1—C1—N1	121.5 (8)	C23—C28—C27	120.7 (10)
O1—C1—C2	121.5 (8)	C23—C28—H28	119.7
N1—C1—C2	117.0 (8)	C27—C28—H28	119.7
C7—C2—C3	118.9 (8)	O5—C29—N5	122.2 (7)
C7—C2—C1	124.6 (9)	O5—C29—C30	120.9 (8)
C3—C2—C1	116.4 (9)	N5—C29—C30	116.9 (8)
C4—C3—C2	121.2 (11)	C35—C30—C31	118.7 (8)
C4—C3—H3A	119.4	C35—C30—C29	118.3 (8)
C2—C3—H3A	119.4	C31—C30—C29	123.0 (8)
C3—C4—C5	121.2 (11)	C32—C31—C30	120.7 (9)
C3—C4—H4A	119.4	C32—C31—H31	119.6
C5—C4—H4A	119.4	C30—C31—H31	119.6
C4—C5—C6	119.9 (11)	C33—C32—C31	120.5 (10)
C4—C5—H5A	120.0	C33—C32—H32	119.7
C6—C5—H5A	120.0	C31—C32—H32	119.7
C5—C6—C7	118.9 (11)	C32—C33—C34	120.4 (10)
C5—C6—H6A	120.5	C32—C33—H33	119.8
C7—C6—H6A	120.5	C34—C33—H33	119.8
C2—C7—C6	119.7 (10)	C33—C34—C35	119.6 (10)
C2—C7—H7	120.2	C33—C34—H34	120.2
C6—C7—H7	120.2	C35—C34—H34	120.2
O2—C8—N2	121.4 (7)	C30—C35—C34	120.0 (10)
O2—C8—C9	122.3 (8)	C30—C35—H35	120.0
N2—C8—C9	116.3 (8)	C34—C35—H35	120.0
C14—C9—C10	120.1 (8)	O6—C36—N6	120.5 (8)
C14—C9—C8	117.3 (9)	O6—C36—C37	121.5 (8)
C10—C9—C8	122.6 (8)	N6—C36—C37	118.0 (9)
C9—C10—C11	120.1 (8)	C42—C37—C38	117.1 (9)
C9—C10—H10	120.0	C42—C37—C36	118.7 (9)
C11—C10—H10	120.0	C38—C37—C36	124.2 (9)
C12—C11—C10	119.8 (10)	C39—C38—C37	120.6 (11)
C12—C11—H11	120.1	C39—C38—H38	119.7
C10—C11—H11	120.1	C37—C38—H38	119.7
C13—C12—C11	119.0 (9)	C38—C39—C40	120.4 (13)
C13—C12—H12	120.5	C38—C39—H39	119.8
C11—C12—H12	120.5	C40—C39—H39	119.8
C12—C13—C14	122.2 (10)	C41—C40—C39	119.7 (13)
C12—C13—H13	118.9	C41—C40—H40	120.2
C14—C13—H13	118.9	C39—C40—H40	120.2
C9—C14—C13	118.8 (10)	C42—C41—C40	119.5 (13)
C9—C14—H14	120.6	C42—C41—H41	120.3
C13—C14—H14	120.6	C40—C41—H41	120.3
O3—C15—N3	123.3 (7)	C41—C42—C37	122.6 (12)
O3—C15—C16	120.3 (8)	C41—C42—H42	118.7
N3—C15—C16	116.4 (8)	C37—C42—H42	118.7
C17—C16—C21	117.0 (9)	N7—C43—O7	113 (4)
C17—C16—C15	124.9 (8)	N7—C43—H43	123.4
C21—C16—C15	118.0 (8)	O7—C43—H43	123.4
C16—C17—C18	122.8 (11)	N7—C44—H44A	109.5
C16—C17—H17	118.6	N7—C44—H44B	109.5
C18—C17—H17	118.6	H44A—C44—H44B	109.5
C19—C18—C17	120.7 (12)	N7—C44—H44C	109.5
C19—C18—H18	119.6	H44A—C44—H44C	109.5
C17—C18—H18	119.6	H44B—C44—H44C	109.5
C18—C19—C20	119.9 (11)	N7—C45—H45A	109.5
C18—C19—H19	120.1	N7—C45—H45B	109.5
C20—C19—H19	120.1	H45A—C45—H45B	109.5
C19—C20—C21	118.8 (12)	N7—C45—H45C	109.5
C19—C20—H20	120.6	H45A—C45—H45C	109.5
C21—C20—H20	120.6	H45B—C45—H45C	109.5

### Hydrogen-bond geometry (Å, °)

**Table:**

*D*—H···*A*	*D*—H	H···*A*	*D*···*A*	*D*—H···*A*
N1—H1···O5^i^	0.86	2.04	2.846 (9)	156
N6—H6···O2^ii^	0.86	2.01	2.826 (9)	157
N2—H2···O3	0.86	2.00	2.800 (9)	155
N3—H3···O6	0.86	1.96	2.778 (10)	158
N4—H4···O1	0.86	1.92	2.743 (9)	160
N5—H5···O4	0.86	1.97	2.774 (9)	155

Symmetry codes: (i) *x*, *y*+1, *z*; (ii) *x*, *y*−1, *z*.
